# Quest for a summary measure of biological age: the health and retirement study

**DOI:** 10.1007/s11357-021-00325-1

**Published:** 2021-02-05

**Authors:** Eileen M. Crimmins, Bharat Thyagarajan, Jung Ki Kim, David Weir, Jessica Faul

**Affiliations:** 1 Davis School of Gerontology, University of Southern California, Los Angeles, CA USA; 2 University of Minnesota, Minneapolis, MN USA; 3 Institute for Social Research, University of Michigan, Ann Arbor, MN USA

**Keywords:** Biological age, Phenotypic age, TAME markers, Biomarkers

## Abstract

Measures of biological age and its components have been shown to provide important information about individual health and prospective change in health as there is clear value in being able to assess whether someone is experiencing accelerated or decelerated aging. However, how to best assess biological age remains a question. We compare prediction of health outcomes using existing summary measures of biological age with a measure created by adding novel biomarkers related to aging to measures based on more conventional clinical chemistry and exam measures. We also compare the explanatory power of summary biological age measures compared to the individual biomarkers used to construct the measures. To accomplish this, we examine how well biological age, phenotypic age, and expanded biological age and five sets of individual biomarkers explain variability in four major health outcomes linked to aging in a large, nationally representative cohort of older Americans. We conclude that different summary measures of accelerated aging do better at explaining different health outcomes, and that chronological age has greater explanatory power for both cognitive dysfunction and mortality than the summary measures. In addition, we find that there is reduction in the variance explained in health outcomes when indicators are combined into summary measures, and that combining clinical indicators with more novel markers related to aging does best at explaining health outcomes. Finally, it is hard to define a set of assays that parsimoniously explains the greatest amount of variance across the range of health outcomes studied here. All of the individual markers considered were related to at least one of the health outcomes.

**Supplementary Information:**

The online version contains supplementary material available at 10.1007/s11357-021-00325-1.

## Introduction

It is generally accepted that chronological age is a less than perfect indicator of the health of aging individuals. Determining the physiological changes linked to age and related to health outcomes for individuals provides important information about influences on the aging process as well as information on potential areas for intervention to delay the process of health change with age.

A variety of approaches to measuring biological age using widely varying sets of biomarkers and summary measures have been suggested in recent years. The desire to combine indicators into summary measures that better predict health outcomes originated with the Framingham Risk Score [1]. Subsequently, a body of work demonstrated that multiple health outcomes common at older ages were related to multisystem dysregulation as measured by allostatic load [2]. Measures of biological age were developed in response to developments in “Geroscience” that emphasizes a set of underlying mechanisms reflecting aging of the organism [3–5], as well as in response to a desire to intervene in the aging process [6]. In general, summary indices of biological aging based on clinical or biochemical indicators have done somewhat better in explaining health outcomes among aging populations than molecular or cellular measures that are thought to reflect the basic mechanisms of aging [3, 7]. This may be because the clinical and biochemical indicators capture downstream physiological change that is more closely linked to health outcomes. Here we attempt to define a limited number of clinical or biochemical indicators that explain a variety of major health outcomes linked to age. If the set of markers explains the health outcomes equally well, this would provide support for the basic tenet of Geroscience that one underlying process is responsible for multiple health outcomes [4]. We examine both summary measures of biological aging and individual markers and include some novel age-related markers. Clarifying the links between health outcomes and individual biomarkers as well as summary measures based on multiple indicators will be informative for those undertaking data collection for both clinical and observational studies who have limited resources and need to choose among biomarkers.

While it is hypothesized that all age-related health outcomes reflect the same basic aging processes, it is not clear that all aging-related health outcomes relate to clinical markers in the same way. Generally, research has evaluated links between summary biological age measures and a limited number of outcomes at any one time including mortality [8], ADLs and IADLs and mortality [9], and disease onset and mortality [10]. One set of biomarkers may not do equally well in predicting multiple health outcomes because they may be differentially related to the physiological systems indexed by the biomarkers. Testing a range of outcomes that cover the morbidity process in one study will provide an indication of whether we are capturing one process that affects all outcomes.

In this paper, we address the question of how suggested summary measures of biological age and individual biomarkers characterize risk for major aging-related health outcomes. We examine multiple approaches to measuring biological aging using 24 biomarkers grouped in multiple ways. Some of the biomarkers are fairly common clinical indicators and some are more novel measures collected specifically because they are thought to be important in the aging process and have been suggested by experts in aging. We relate the biomarkers to four major health outcomes linked to aging; and we examine how well the variability in the health outcomes is explained by three summary measures of biological age and the individual markers in the summary measures. In addition, we indicate how much additional explanatory power is provided by the biological measures compared to chronological age. Our hypothesis is that including novel biomarkers related to aging and more biomarkers in the measures will improve our ability to explain health outcomes. Finally, we develop a parsimonious set of markers to explain each health outcome to see if the set of reduced markers is similar across health outcomes and thus would suggest collecting data on a more parsimonious set of measures. We use data from a large nationally representative sample of the US population over the age of 56 in the analysis.

## Data and measures

### Health and retirement study

The data come from the nationally representative Health and Retirement Study (HRS), a biennial longitudinal study of the population over age 50. The biomarker data were collected in 2016 from 9193 respondents who completed the 2016 interview themselves, for whom this was not their baseline interview, who were not in a nursing home, and who agreed to and completed a subsequent blood collection. The blood collection producing the values for most of the biomarkers was done by phlebotomists and took place in respondents’ homes about 2 months after the 2016 interview. Blood was centrifuged in the field and sent cold to the Advanced Research & Diagnostics Laboratory at the University of Minnesota, arriving within 24 h for most samples (details are available at the Health and Retirement Study website [11]). The samples were assayed in this lab for biomarkers thought to be related to aging. Three measures used in the analysis (blood pressure, peak flow, and HbA1c) come from data collected in the home by interviewers in half of the samples in 2014 and 2016 because HbA1c was not assayed in the 2016 blood collection but was one of the assays done using dried blood spots in 2014 and 2016.

The sample used in this analysis is 4287 persons between age 56 and 90. There are missing cases in some of the variables. The sample in this analysis does not differ from the initial sample of 9193 in age and sex, but has somewhat fewer health problems (see Supplementary Table 1). Most of these differences are small; there is, however, a large difference in the percent who died in the two samples: only 2.6% in the included sample and 4.6% in the omitted sample. Since the mortality was on average only 1 year after the blood collection, many of these people may already have been ill and not willing to give blood. The analysis uses weights developed by HRS for the biomarker sample so that results reflect those for a national sample.

### Biological measures

The biological measures examined here are 24 individual markers included in biological age [8], phenotypic age [12], and the TAME assays [13]. The three summary measures examined are biological age, phenotypic age, and an expanded biological age measure that includes all of the assays in the 3 groups except for two highly co-linear measures of creatinine and fasting glucose. Biological age, phenotypic age, and the TAME list of assays were developed using different approaches, different methods, and for different uses although all are attempts to characterize biological aging.

“Biological age” was developed to serve as an overall measure of an individual’s physiological status that is used to define whether aging was accelerated or delayed relative to chronological age [8]. In its original formulation, biological age was based on 10 exam or clinical chemistry measures selected out of 25 available in the National Health and Nutrition Examination Survey (NHANES) data. The original selection was based on the correlation between each measure and age among people in the 30 to 75 age range. In this analysis, we use the same 10 measures but we recalibrate the weights for the HRS sample age 56 to 90 and a more current time period as the parameters developed in the NHANES analysis were based on entering the sample from 1988 to 1994 (details shown in the Supplementary Table 2). The biomarkers included in this measure are shown in Table 1. This set of markers includes fairly standard clinical markers that would be collected on many people in a clinical exam: a cardiovascular indicator (systolic blood pressure), metabolic markers (total cholesterol, fasting glucose), markers of inflammation and infection (cytomegalovirus, C-reactive protein, albumin), and markers of organ functioning (serum creatinine, alkaline phosphatase, BUN, peak flow). Use of this measure has been shown to explain mortality differences between African Americans and non-Hispanic whites in the USA and be related to health outcomes in a younger population in New Zealand [14, 15].

**Table 1:** Twenty-four individual biomarkers included in summary measures—biological age and phenotypic age—or the TAME list of assays

	Biological age	Phenotypic age	TAME	Expanded biological age	Mean (SD)	Correlation with age
Systolic blood pressure (mmHg)^1,2^	X			X	127.60 (17.99)	0.17***
Total cholesterol (mg/dL)^2^	X			X	192.25 (41.26)	−0.18***
CMV (COI)^2^	X			X	288.48 (345.40)	0.06***
Serum creatinine (mg/dL)	X	X			0.91 (0.26)	0.20***
Alkaline phosphatase (U/L)^2^	X	X		X	79.45 (25.53)	−0.04**
BUN (mg/dL)^2^	X			X	17.33 (5.76)	0.25***
Peak flow (L/min)^1,2^	X			X	365.74 (131.97)	−0.30***
Albumin (g/dL)	X	X		X	3.99 (0.30)	−0.15***
Fasting glucose (mg/dL)^2^	X	X			104.80 (29.70)	0.00
CRP (mg/L) (logged)	X	X	X	X	−1.51 (0.93)	−0.04**
Lymphocyte %^2^		X		X	30.07 (8.43)	−0.18***
Mean cell volume^2^		X		X	93.20 (5.84)	0.10***
Red cell distribution width		X		X	13.89 (1.26)	0.13***
White blood cell count^2^		X		X	6.57 (1.80)	0.01
IL-6 (pg/mL) (logged)			X	X	1.34 (0.75)	0.16***
TNFRI (pg/mL)^2^			X	X	1733.12 (681.48)	0.32***
IGF 1 (ng/mL)^2^			X	X	106.22 (36.26)	−0.21***
Cystatin C (mg/L)			X	X	1.12 (0.33)	0.39***
NT-proBNP (pg/mL) (logged)			X	X	4.56 (1.18)	0.48***
HbA1c (%)^1^			X	X	5.88 (0.92)	0.07***
IL-10 (pg/mL)^2^			(X)	X	3.67 (1.62)	0.09***
IL-1Ra (pg/mL)^2^			(X)	X	563.82 (321.79)	−0.02
TGFB (pg/mL)^2^			(X)	X	48,285.23 (13,209.71)	−0.22***
CD4/CD8 count^2^			(X)	X	3.87 (2.66)	0.00

All markers are top coded at the 99%ile

^1^2014 and 2016 combined

^2^Due to small but significant effect of these markers on health outcomes in regression equations, we divide these markers by 100 to visualize the effects of these markers on health outcomes in later analyses

***p < .001; **p < .01

The second measure is “phenotypic age” based on 9 measures which Levine and a group of scholars developed specifically to provide a set of markers important in predicting mortality [12]. Phenotypic age represents the age in the population that corresponds with a person’s mortality risk. Calculation of phenotypic age is based on parametrization of 2 Gompertz proportional hazard models—one fit using the 10 selected variables, and the other fit using only chronological age—using data on the US National Health and Nutrition Examination Survey (NHANES) for those 20 to 85+. Out of the 9 measures in phenotypic age, 5 overlap with biological age. Four additional biomarkers assessed in complete blood counts are included with phenotypic age: percent lymphocytes, mean cell volume, red cell distribution width, and white blood cell count.

The third set of measures we investigate is called the Targeting Aging with Metformin or TAME assays [13]. These were selected by a group of scholars in biology of aging and geriatrics as the most important assays to be included in clinical trials aimed at delaying aging with immediate application in a trial of metformin as the aging-delaying drug. Since metformin is a drug developed to treat diabetes, selection of the markers took this into account. The group had extensive weekly discussions occurring over a 6-month period beginning with initial consideration of 258 assays. Nine assays were selected for inclusion in the Core TAME assay list: IL-6, TNF-α receptor I or II, CRP, GDF15, insulin, IGF 1, cystatin C, NT-proBNP, and hemoglobin A1c. We include seven of these as insulin and GDF15 are not available in the data set. We also include expanded markers suggested by the TAME group who indicated that, where reliable laboratories were used on well-collected data, expanded measures could include more cytokines and results from flow cytometry. We include three extra cytokines: IL-10, TGF beta, and IL-1Ra. In addition, we include the CD4/CD8 count, an indicator of immune senescence, from flow cytometry. Only one of the TAME biomarkers overlaps with measures in either of the other two measures—C-reactive protein (CRP)—and this is the one biomarker in all three measures. The TAME assays are recognized generally as biomarkers related to aging, IGF 1, CD4/CD8; to inflammation, the cytokines and CRP; to heart failure, NT-proBNP; to kidney functioning, cystatin C; and to metabolic functioning, HbA1c. This group did not suggest combining these measures into a summary indicator of biological age.

The expanded biological age that we construct uses all the measures in the three suggested summary measures except glucose and serum creatinine. Cystatin C is regarded as an alternative and better research measure than serum creatinine as they both indicate kidney problems and are highly correlated [16]. HbA1c is fairly similar to fasting glucose in its role but because it does not require fasting it is an easier measure to obtain than fasting glucose. We use methods for estimating expanded biological age that are similar to those used to develop the original biological age measure [8].

These summary measures expressed in years can be compared to chronological age and aging can be characterized as accelerated or delayed. We regress each summary measure on age and use the residuals as indicators of accelerated aging or delayed aging. Our regression analysis of the summary measures uses accelerated biological aging, accelerated phenotypic aging, and accelerated expanded biological aging in the analysis.

Because the summary measures were developed with weighting reflecting the links either between individual markers and age or between the markers and mortality, we also examine regressions including the ten, nine, and 22 individual markers in the summary measures, and the eleven TAME markers. In addition, we estimate for each health outcome a parsimonious set of markers based on elastic net regression. These reduced sets of markers provide essentially the same predictive power as the 22 markers. We provide results with the individual measures as well as the summary measures because of our interest in assessing the value of individual components of measures in predicting health outcomes and the relative ability of summary measures and individual markers to explain the variability in age-related health outcomes.

### Health outcomes

We examine the links between the biological measures and four health outcomes: multimorbidity, physical functioning loss, cognitive dysfunction, and mortality. Multimorbidity and physical functioning are reported in the 2018 interview, 2 years after the biological measures. Mortality occurs between 2016 and 2018. The cognitive dysfunction measure is from 2016, the same time as the biology. Multimorbidity ranges from 0 to 5 and reflects the number of doctor diagnosed diseases out of five that an individual reports he/she has in 2018: diabetes, heart disease, lung disease, stroke, and cancer. Loss of physical functioning is indexed by the number of difficulties with performing ADL and IADL tasks (0–10). Cognitive dysfunction is based on a summary cognitive score (0–27) reflecting performance on a series of cognitive tests [17]. It is reversed so that high scores reflect worse functioning.

### Other variables

We include age and sex in equations so results indicate the association of biological variables with health outcomes with these controlled.

## Statistical analysis

First, we examine the correlation of each of the biological markers with age and the intercorrelations among the biomarkers. Then, we examine associations of the biological variables—both summary measures and individual biomarkers—with health outcomes while controlling for sex. Ordinary least squares regression is used to relate biomarkers to three health outcomes: deficits in physical functioning, multimorbidity, and cognitive dysfunction. Logistic regression is used in regressions with mortality. We estimate eight regressions of biological measures for each health outcome: accelerated biological age, accelerated phenotypic age, and accelerated expanded biological age. Then, we examine regressions with each of the individual biological markers in each of these summary measures, the individual TAME assays, and a more parsimonious set of markers for each health outcome chosen from the entire set based on elastic net regression using STATA. We examine the adjusted *R*^2^ for each of the equations with only age and sex controlled at first, and then with the biomarkers to see how well the biomarkers do at explaining the various health outcomes.

## Results

### Sample characteristics

The sample averaged 68 years of age and ranged from 56 through 90 (Table 2). On average, the sample had difficulty with less than 1 ADL/IADL (0.54), and less than one of the 5 diseases (0.84). The average cognitive dysfunction score was 11.25 out of a maximum of 27. Less than 3% (2.6%) of the sample died in 2 years.

**Table 2:** Sample characteristics, the Health and Retirement Study (*N* = 4287; *N* = 3876 for ADL/IADL difficulties and multimorbidity)

	Mean	SD	Range	Percent
Mean age	68.07	8.28	56–90	
%Female				55.5%
Health outcomes				
Mean ADL/IADL difficulties 2018	0.54	1.42	0–10	
Mean number of diseases 2018	0.84	0.95	0–5	
Mean cognitive dysfunction 2016	11.25	4.29	0–27	
% Mortality in 2 years—2016–2018				2.6%
Biological age	67.94	9.68	44.52–109.71	
Accelerated biological age^1^	0.00	5.11	−16.64-36.39	
Phenotypic age	67.51	12.22	41.52–121.58	
Accelerated phenotypic age^1^	0.00	8.28	−20.01-62.85	
Expanded biological age	68.06	11.34	43.43–119.54	
Accelerated expanded biological age^1^	0.00	7.77	−20.01-50.63	

^1^Accelerated age is defined as residual from a regression of the summary measures of biological age, phenotypic age, and expanded biological age on chronological age

The average biological age of the sample was 68, identical to the chronological age by design. The range of accelerated biological age was minus 17 to plus 36. The means of phenotypic age and expanded biological age were similar to biological age, 68. Both of these measures had a wider range of values (accelerated phenotypic age, from − 20 to 63; and expanded biological age, from − 20 to 51). While the distributions of all three summary measures are fairly normal, they all have a right tail. The highest value of phenotypic age (121.58) is just below that of Jeanne Calment recognized as the longest lived human (122.45 years).

### Links of biomarkers to age

Summary measures of biological age, phenotypic age, and expanded biological age were all highly correlated with age (0.85, 0.74, and 0.73, respectively). They were also strongly correlated with each other: biological age and phenotypic age (0.79), biological age and expanded biological age (0.90), and phenotypic age and expanded biological age (0.82). All but 4 of the individual biomarkers were correlated with age: fasting glucose, white blood cell count, IL-1Ra, and the CD4/CD8 count (Table 1). The strongest associations of age were with cystatin C (0.39), NT-proBNP (0.48), and TNFRI (0.32). Total cholesterol, CRP, and alkaline phosphatase were negatively associated with age which is somewhat surprising. Interestingly, very few biomarkers were related exponentially to age as most did not have a significant coefficient on age^2^ when this was examined (Supplementary Table 3); however, this is after several variables were logged because of their distribution.

### Links between the biomarkers

**Fig. 1:**
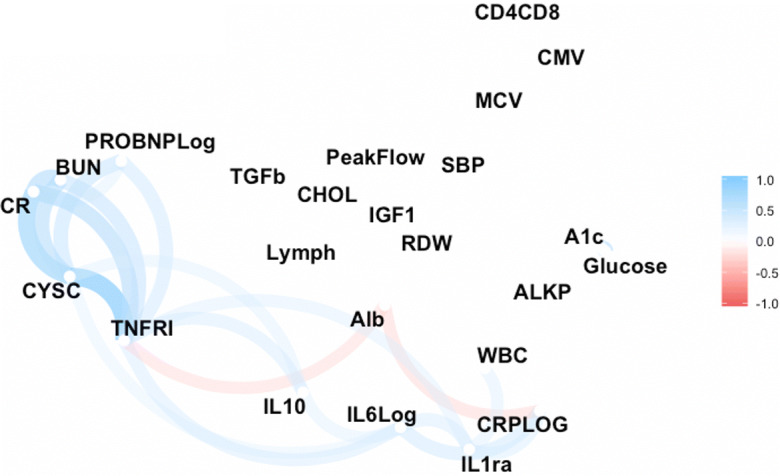
A Correlation Network map among 24 biomarkers

Most of the markers did not relate highly to each other (Supplementary Table 3 and Fig. 1). The two markers that measure similar physiology had the highest relationship: cystatin C and serum creatinine (0.72), and HbA1c and fasting glucose (0.61). The strength of the correlations among markers is shown in Fig. 1, indicating the Correlation Network in graphic form. This figure groups more highly related markers and darker colors indicate stronger relationships. Relationships above 0.3 are shown in the figure. The light color of the figure and the spread indicate that most markers were not highly related. The figure clarifies that trying to make all the measures into “factors” does not seem appropriate as they do not load onto a small number of factors, but at first pass have 8 factors. This is an additional reason we provide analysis with the individual markers as well as the summary measures.

### How well do three summary measures of accelerated aging explain four health outcomes?

Equations with the results of the regressions of accelerated biological age, accelerated phenotypic age, and accelerated expanded biological age on the four health outcomes are shown in Table 3. The three summary measures of biology were significantly related to each of the outcomes as predicted although the amount of variance explained in the outcomes is limited. Since all three were measured in years, one can compare the coefficients and the *R*^2^ for the equations. Problems with ADL and IADL functioning were similarly explained by the three summary measures but the highest adjusted *R*^2^ is less than 5%. Accelerated biological age was somewhat stronger in its association with cognitive dysfunction with a larger coefficient and more variance explained although the explanatory power of all the summary measures was again relatively low, with sex included in the equation, Accelerated biological age explained 6% of the variance versus 2–4% for the others. About twice as much of the variability in multimorbidity was explained by accelerated phenotypic aging (10%) and accelerated expanded biological age (9%) than by accelerated biological age (5%). Variance in mortality was best explained by the accelerated expanded biological age (11%).

**Table 3:** Coefficients from regressions of chronological age, accelerated biological age, accelerated phenotypic age, and accelerated expanded biological age on ADL/IADL difficulties, multimorbidity, cognitive dysfunction, and odds ratios from regressions of mortality (*N* = 4287; *N* = 3876 for ADL/IADL difficulties and multimorbidity)

	Chronological age	Accelerated biological age	Accelerated phenotypic age	Accelerated expanded biological age
ADL/IADL difficulties				
Age	0.03***			
Accelerated biological age		0.05***		
Accelerated phenotypic age			0.03***	
Accelerated expanded biological age				0.04***
Female	0.11*	0.12**	0.19***	0.12**
Adj *R*^2^	0.0258	0.0330	0.0328	0.0474
Multimorbidity				
Age	0.03***			
Accelerated biological age		0.04***		
Accelerated phenotypic age			0.04***	
Accelerated expanded biological age				0.04***
Female	−0.10***	−0.09**	−0.01	−0.09**
Adj *R*^2^	0.0584	0.0477	0.0968	0.0871
Cognitive dysfunction				
Age	0.16***			
Accelerated biological age		0.20***		
Accelerated phenotypic age			0.08***	
Accelerated expanded biological age				0.11***
Female	−0.20	−0.12	0.05	−0.12
Adj *R*^2^	0.0958	0.0561	0.0204	0.0381
Mortality between 2016 and 2018				
Age	1.12***			
Accelerated biological age		1.14***		
Accelerated phenotypic age			1.07***	
Accelerated expanded biological age				1.10***
Female	0.84	1.07	1.07	0.97
Pseudo-*R*^2^	0.1208	0.0811	0.0719	0.1128

***p < .001; **p < .01; *p < .05

We also include regression results from an equation with chronological age and sex as the independent variables in Table 3. This allows us to compare the variance explained by age and the summary biological measures. Chronological age does better at explaining cognitive functioning problems and mortality than the summary measures. Accelerated phenotypic age and accelerated expanded biological age provide enhanced explanation of multimorbidity over that provided by chronological age.

### How well do five sets of individual biomarkers explain four health outcomes?

Because we want to compare the set of TAME measures with the biomarkers in the summary measures, we related the individual biomarkers in biological age, phenotypic age, the TAME assays, and expanded biological age to the health outcomes. We also included a more parsimonious set of biomarkers for each outcome chosen using elastic net regression. Tables 4, 5, 6, and 7 provide the details of the relationships between individual biomarkers and health outcomes.

**Table 4:** Coefficients from regressions of ADL/IADL difficulties on age, and individual markers in biological age, phenotypic age, TAME assays, expanded biological age, and parsimonious biological age for ADL/IADL difficulties (*N* = 3876)

	W/ age + sex	W/ biological age markers	W/ phenotypic age markers	W/ TAME assays	W/ expanded biological age markers	Parsimonious biological age 11 markers
Systolic blood pressure^1^		0.20			0.07	
Total cholesterol		−0.0			0.08	0.12
CMV		0.01			−0.00	
Serum creatinine		0.16	0.04			
Alkaline phosphatase		0.41***	0.29***		0.31**	0.33***
BUN		0.30			−0.82	
Peak flow^1^		−0.23***			−0.19***	−0.20***
Albumin		−0.43**	−0.16*		−0.29***	−0.28***
Fasting glucose		0.16*	0.03			
CRP (logged)		0.06*	0.07**	0.07*	0.00	
Lymphocyte %			0.56*		0.99***	
Mean cell volume			−0.33		−0.98*	
Red cell distribution width			0.08***		0.08***	0.09***
White blood cell count			5.18***		2.97*	2.38
IL-6 (logged)				0.13***	0.09*	0.05
TNFRI				−0.00	0.00	
IGF 1				−0.03	0.05	
Cystatin C				0.49***	0.45***	0.32***
NT-proBNP (logged)				0.13***	0.11***	0.10***
HbA1c^1^				0.11***	0.06*	0.06*
IL-10				−1.39	−0.87	
IL-1Ra				−0.01	−0.01	
TGFB				0.00**	0.00	
CD4/CD8				−0.99	−0.20	−0.51
Age	0.03***	0.01***	0.03***	0.01**	0.01*	0.00
Female	0.11*	−0.24***	0.07	0.07	−0.28***	−0.24***
Adj *R*^2^	0.0258	0.0846	0.0755	0.0748	0.1111	0.1067

^1^2014 and 2016 combined

***p < .001; **p < .01; *p < .05

**Table 5:** Coefficients from regressions of multimorbidity on age, and individual markers in biological age, phenotypic age, TAME assays, expanded biological age, and parsimonious biological age for multimorbidity (*N* = 3876)

	W/ age + sex	W/ biological age markers	W/ phenotypic age markers	W/ TAME assays	W/ expanded biological age markers	Parsimonious biological age 17 markers
Systolic blood pressure^1^		−0.04			−0.12	−0.11
Total cholesterol		−0.42***			−0.29***	−0.29***
CMV		−0.00			−0.00	
Serum creatinine		0.39***	0.33***			
Alkaline phosphatase		0.12*	0.16*		0.07	
BUN		−0.36			−0.64*	
Peak flow^1^		−0.13***			−0.09***	−0.09***
Albumin		−0.01	−0.11*		0.10*	0.10
Fasting glucose		0.89***	0.94***			
CRP (logged)		0.08***	0.04*	0.03	0.04*	0.05**
Lymphocyte %			−0.36*		−0.34*	−0.35*
Mean cell volume			0.22		0.28	0.26
Red cell distribution width			0.06***		0.02	0.02
White blood cell count			2.91***		0.62	
IL-6 (logged)				0.03	0.00	0.01
TNFRI				0.00	0.00	
IGF 1				0.07	0.09*	0.08*
Cystatin C				0.16*	0.20*	0.17**
NT-proBNP (logged)				0.14***	0.12***	0.12***
HbA1c^1^				0.35***	0.33***	0.33***
IL-10				1.01	0.85	0.95
IL-1Ra				0.02***	0.01**	0.02**
TGFB				−0.00	−0.00	−0.00
CD4/CD8				−1.00*	−1.01*	−1.02*
Age	0.03***	0.02***	0.02***	0.01***	0.01***	0.01***
Female	−0.10**	−0.11**	−0.00	−0.13***	−0.19***	−0.19***
Adj *R*^2^	0.0584	0.2281	0.1934	0.2487	0.2717	0.2712

^1^2014 and 2016 combined

***p < .001; **p < .01; *p < .05

**Table 6:** Coefficients from regressions of cognitive dysfunction on age, and individual markers in biological age, phenotypic age, TAME assays, and expanded biological age (*N* = 4287)

	W/ age + sex	W/ biological age markers	W/ phenotypic age markers	W/ TAME assays	W/ expanded biological age markers
	M1	M1	M1	M1	M1
Systolic blood pressure^1^		0.90**			0.77*
Total cholesterol		−0.14			−0.02
CMV		0.07***			0.04*
Serum creatinine		1.16***	0.91***		
Alkaline phosphatase		0.59*	0.99***		0.46
BUN		−2.03			−0.69
Peak flow^1^		−1.15***			−1.09***
Albumin		−0.88***	−1.13***		−0.69**
Fasting glucose		0.77***	0.86***		
CRP (logged)		0.01	0.11	0.12	−0.06
Lymphocyte %			4.24***		3.04***
Mean cell volume			−1.94		−2.30*
Red cell distribution width			0.18***		0.09
White blood cell count			14.24***		6.46
IL-6 (logged)				0.38***	0.25*
TNFRI				−0.02	−0.03
IGF 1				−0.61***	−0.36*
Cystatin C				1.38***	1.09**
NT-proBNP (logged)				0.24***	0.09
HbA1c^1^				0.45***	0.25***
IL-10				3.76	6.93
IL-1Ra				−0.06*	−0.04*
TGFB				0.00*	0.00
CD4/CD8				−9.10***	−4.56*
Age	0.16***	0.09***	0.16***	0.12***	0.09***
Female	−0.20	−1.75***	−0.21	−0.26*	−1.96***
Adj *R*^2^	0.0958	0.2031	0.1332	0.1354	0.2124

^1^2014 and 2016 combined

***p < .001; **p < .01; *p < .05

**Table 7:** Odds ratios from regressions of mortality on age and individual biomarkers in biological age, phenotypic age, TAME assays, expanded biological age, and parsimonious biological age markers for mortality (*N* = 4287)

	W/ age + sex	W/ biological age markers	W/ phenotypic age markers	W/ TAME assays	W/ expanded biological age markers	Parsimonious biological age 10 markers
Systolic blood pressure^1^		2.35			2.16	2.56
Total cholesterol		1.04			1.45	
CMV		1.03			1.04	
Serum creatinine		2.21	3.22***			
Alkaline phosphatase		1.88	2.09*		1.76	
BUN		33.65			1.70	
Peak flow^1^		0.54***			0.59***	0.57***
Albumin		0.86	0.90		1.14	
Fasting glucose		1.67	1.85*			
CRP (logged)		1.19	1.15	1.11	0.99	
Lymphocyte %			0.02**		0.07	0.08
Mean cell volume			76.31**		47.71*	22.77
Red cell distribution width			1.33***		1.29***	1.28***
White blood cell count			–		256.48	
IL-6 (logged)				1.23	1.03	1.02
TNFRI				1.02	1.01	1.00
IGF 1				1.21	1.12	
Cystatin C				2.32*	1.88	2.08
NT-proBNP (logged)				1.66***	1.48***	1.41*
HbA1c^1^				1.04	1.04	
IL-10				1.82	11.05	4.69
IL-1Ra				0.99	0.98	
TGFB				1.00**	1.00	
CD4/CD8				0.17	0.95	
Age	1.12***	1.08***	1.10***	1.07***	1.06***	1.05***
Female	0.84	0.48**	1.14	0.79	0.40***	0.43***
Adj *R*^2^	0.1208	0.2290	0.2102	0.2342	0.2886	0.2760

^1^2014 and 2016 combined;

***p < .001; **p < .01; *p < .05

The adjusted *R*^2^ is substantially higher in these equations containing individual markers than in the equations presented in Table 3 based on the summary measures. For instance, the pseudo-*R*^2^ is 11% in the mortality equation with accelerated expanded biological age and 29% using the individual markers in expanded biological age. For each outcome, the equations with the expanded set of 22 biomarkers resulted in the highest adjusted *R*^2^. The TAME assays came close to the explanatory power of the 22 assays in the case of multimorbidity (25% versus 27%). For the other 3 health outcomes, the assays in biological age were second to the expanded list of assays in explaining variance. The best equations explained about 20–30% of the total variability in the outcomes: multimorbidity, cognitive dysfunction, and mortality. The explanation of the variability in ADL and IADL functioning was much less, maximal 11%.

Using elastic net regression, we identified subsets of the list of 22 biomarkers that did approximately as well as the 22 markers in explaining three of the health outcomes. These included 11, 17, and 10 biomarkers for ADL/IADL difficulties, multimorbidity, and mortality, respectively. A smaller set of variables could not be identified for cognitive dysfunctioning. The significance of individual markers along with their coefficients is shown in Tables 4, 5, 6, and 7. There was no marker that never had a significant effect on some outcome; whether a marker was significant often depended on what other markers were in the equation (e.g., CRP). When all of the markers in the biological age, phenotypic age, TAME assays, and expanded biological age were in the equations at one time, only some were significant in explaining any outcome. There were three of the markers from the parsimonious equations that had significant effects in the expected direction on at least three health outcomes: peak flow, cystatin C, and NT-proBNP (Tables 4, 5, 6, and 7, summarized in Supplementary Table 5). It is also noteworthy that four individual biomarkers (albumin, lymphocyte percent, IGF 1, and IL-1Ra) had significant effects on cognitive dysfunction and multimorbidity but in each case one of the relationships was in the unexpected direction. This is, of course, when many other biomarkers were in the equation.

## Discussion

We have examined the association of three summary biological age acceleration measures and 24 individual biomarkers with four age-related health outcomes in a large data set representative of all Americans 56 years of age and older. One tenet of Geroscience is that all age-related health outcomes arise from the same underlying processes; but we find that these biomarkers and summary measures are better at explaining some health outcomes than others and that different biomarkers are significantly related to different outcomes. The summary markers of accelerated aging do somewhat better at explaining multimorbidity and do not explain much of the variability in physical or cognitive functioning. In fact, they are not better than chronological age in explaining cognitive functioning problems and mortality. Our incorporation of cognitive functioning is unique and points to the importance of considering a range of aging outcomes in generalizing about the link between indicators of biological age and health. Of course, none of these biomarkers was chosen because of links to cognitive function and this outcome may require a different set of biomarkers. There may be a small number of underlying processes at the root of all of these aging outcomes, but they may be operating on different aspects of biology and at different speeds and that is what is being captured here.

Parker et al. [9] considered the effects on physical functioning and mortality of three summary measures of biological age including phenotypic age (which they term LM) and biological age (which they term KDM) considered here. They concluded that the results were only modestly affected by the different biomarker sets used to develop the measures and that the biological age generally was a better predictor of health outcomes. We would conclude similarly that there was not much difference in the explanatory power of the biological age and phenotypic age measures on physical functioning and mortality; but we would not conclude this for cognitive functioning or multimorbidity. For mortality, we would also conclude that adding the age-related measures into expanded biological age improved our prediction.

The summary measures are useful as they are easily interpreted and capture indicators of many clinical and physiological changes, but they have lower explanatory power than the original biological measures and they mask some of the details of how individual measures relate to outcomes. As independent variables explaining outcomes, the individual biomarkers appear more useful.

The individual biomarkers included in the three different summary approaches to measuring accelerated aging differ markedly, and, in fact, only CRP was in all three summary measures. When the individual biomarker measures are examined, the 22 assays were best at explaining the variability in all outcomes. Variability in cognitive functioning is generally not well explained by these biomarkers but it is particularly poorly explained by the biomarkers in phenotypic age and the TAME assays.

The outcomes of this analysis point to the value of including large numbers of biomarkers which index many systems and physiological processes including both conventional clinical measures and specific measures indexing the aging process as the expanded list of markers does significantly better at explaining these health outcomes. While it is possible to develop more parsimonious lists of biomarkers that explain individual health outcomes, it is hard to develop a more parsimonious set if one wants to explain health outcomes spanning the range studied here. Our list of biomarkers includes a number of indicators of organ functioning, indicators of blood cell distribution, classical cardiovascular and metabolic indicators, a substantial number of inflammatory markers, and markers which can be categorized as indicators of “aging.” All of them seem to be linked to some health outcomes, but how they link often depends on what other markers and how many other markers are being considered. Thus, the results from studies that include different biomarkers and different numbers of biomarkers may differ. Our results have some similarity to those of an analysis of 74 biomarkers in the Newcastle study of a group above the age of 85 [18]. They examined four similar health outcomes and found only 10 of the biomarkers out of the 74 were significantly associated with at least two of the health outcomes. While many of their identified biomarkers are not measured or are not included in our analysis, our findings indicate that NT-proBNP and lung function are important. Along with cystatin C, these are the indicators that were retained in the parsimonious measures for at least three outcomes in our analysis. On the other hand, Gruenewald et al. [19] used recursive partitioning to link a range of biomarkers to mortality among a sample of people aged 70–79 in three areas of the USA and concluded that neuroendocrine and inflammatory markers were the important predictors. While our analysis does not include neuroendocrine makers, inflammatory markers appear to play a somewhat minor role in mortality explanation in our analysis. Studies with different markers on different populations are hard to integrate into a clear picture on what is important.

Our study has limitations in that we do not include indicators of fundamental molecular and cellular process in summary measures of accelerated aging; included markers reflect more downstream health processes. There are also many other biomarkers that could be considered. In addition, the links between the biomarkers and the health outcomes other than mortality indicate the presence of health problems rather than the onset, which would require a longitudinal analysis over a longer time span. The one outcome that we examine prospectively, mortality, suffers from small numbers and the fact that many of those who will die in the 2 years after the blood collection choose not to provide a blood sample. Future data collection will allow both more indicators of fundamental aging processes and physiological state, and longitudinal analysis over a longer period and the onset of health problems. Finally, our sample is limited in that people in nursing homes were not included in the blood collection and so this segment of the population is not represented.

Even with these limitations, we can conclude that adding additional biomarkers, particularly those thought to be important in the aging process, improves our ability to predict health outcomes in the older population. Our analysis of variability in the links of biomarkers to health outcomes provides useful information for those who may be considering clinical trials related to aging outcome or the collection of data to study age-related health outcomes.

## Supplementary information

ESM 1 (DOCX 917 kb)

## Data Availability

All data on which this analysis is based are publicly available at the Health and Retirement Study website.
