# Supplementary material for: Quest for a summary measure of biological age: the health and retirement study
Source: GeroScience. 2021 Feb 5;43(1):395–408. doi: 10.1007/s11357-021-00325-1 (PMC8050146; doi:10.1007/s11357-021-00325-1)
Supplement: Supplementary file 1 — (DOCX 917 kb) [file 11357_2021_325_MOESM1_ESM.docx]

**Supplementary Information**

**Analysis of Missing Data**

Among the original sample of 9,193 in the HRS 2016 VBS data, we selected those between 56 and 90 years of age (N=9,037). Among them, 5,397 have information on the variables needed for biological age and 3,640 have missing information in one or more of 10 biological markers (fasting glucose has 3,161 missing cases; systolic blood pressure has 725 missing cases; peak flow has 678 missing cases). This is due to the fact that half of the systolic blood pressure and peak flow information is from the 2014 exam; 9,361 have Phenotypic Age; and 5,216 have both Biological Age and Phenotypic Age. Among these, an additional 840 are missing from some of the TAME or expanded markers and 90 are missing from our health outcomes. Thus, our final analytic sample is 4,287 who have all markers of interest.

Those who are missing are not different in age or gender from the analytic sample. Those who are missing have a higher number of ADL/IADL difficulties, more comorbidities, and greater cognitive dysfunction in the 2016 survey. Those missing are more likely to die in 2 years.

**Supplementary Table 1. Characteristics of Analytic Sample and Missing Sample Members**

|  | Non missing | Missing | P value |
| --- | --- | --- | --- |
|  | (N=4,287) | (N=4,750) |  |
| Mean age | 68.1 | 67.6 | NS |
| % Female | 55.5% | 53.8% | NS |
| Mean ADL/IADL difficulties | 0.46 | 0.56 | .0315 |
| Mean number of diseases (multimorbidity) | 0.78 | 0.84 | .0312 |
| Mean cognitive dysfunction | 11.25 | 11.56 | .0371 |
| % Dead | 2.6% | 4.6% | .0008 |
|  |  |  |  |
|  |  |  |  |
|  |  |  |  |

**Supplementary Table 2. Parameters for Estimation of Levine Biological Age**

|  | K (slope) | Q (intercept) | S (RMSE) |
| --- | --- | --- | --- |
| Systolic Blood Pressure (mmHg) | 7.754 | 58.179 | 8.148 |
| Total cholesterol (mg/dL) | -3.693 | 75.174 | 8.125 |
| CMV (COI) | 0.138 | 67.676 | 8.253 |
| Serum Creatinine (mg/dL) | 6.278 | 62.342 | 8.101 |
| Alkaline phosphatase (U/L) | -1.309 | 69.118 | 8.261 |
| BUN (mg/dL) | 36.224 | 61.795 | 8.000 |
| Peak Flow (L/min) | -1.880 | 74.949 | 7.886 |
| Albumin (g/dL) | -4.164 | 84.670 | 8.172 |
| Fasting glucose (mg/dL) | 0.129 | 67.938 | 8.267 |
| CRP (logged) (mg/L) | -0.335 | 67.567 | 8.263 |

**Parameters for Estimation of Phenotypic Age**

|  | K (slope) | Q (intercept) | S (RMSE) |
| --- | --- | --- | --- |
| Serum Creatinine (mg/dL) | 6.278 | 62.342 | 8.101 |
| Alkaline phosphatase (U/L) | -1.309 | 69.118 | 8.261 |
| Albumin (g/dL) | -4.164 | 84.670 | 8.172 |
| Fasting glucose (mg/dL) | 0.129 | 67.938 | 8.267 |
| CRP (logged) (mg/L) | -0.335 | 67.567 | 8.263 |
| Lymphocyte (%) | -17.220 | 73.250 | 8.138 |
| Mean Cell Volume | 14.773 | 54.304 | 8.221 |
| Red Cell Distribution Width | 0.869 | 55.999 | 8.194 |
| White Blood Cell Count | 2.415 | 67.914 | 8.266 |

**Parameters for Estimation of TAME Assays**

|  | K (slope) | Q (intercept) | S (RMSE) |
| --- | --- | --- | --- |
| CRP (logged) (mg/L) | -0.335 | 67.567 | 8.263 |
| IL6 (logged) | 1.815 | 65.634 | 8.153 |
| TNFRI | 0.393 | 61.255 | 7.820 |
| IGF1 | -4.704 | 73.070 | 8.088 |
| Cystatin C | 9.668 | 57.224 | 7.614 |
| NT-PROBNP (logged) | 3.373 | 52.687 | 7.242 |
| HbA1c | 0.651 | 64.245 | 8.244 |
| IL-10 | 45.991 | 66.386 | 8.233 |
| IL-1Ra | -0.039 | 68.293 | 8.265 |
| TGFB | -0.014 | 74.841 | 8.056 |
| CD4/CD8 | 0.306 | 68.061 | 8.266 |

**Parameters for Estimation of Expanded Biological Age**

|  | K (slope) | Q (intercept) | S (RMSE) |
| --- | --- | --- | --- |
| Systolic Blood Pressure (mmHg) | 7.754 | 58.179 | 8.148 |
| Total cholesterol (mg/dL) | -3.693 | 75.174 | 8.125 |
| CMV (COI) | 0.138 | 67.676 | 8.253 |
| Alkaline phosphatase (U/L) | -1.309 | 69.118 | 8.261 |
| Albumin (g/dL) | -4.164 | 84.670 | 8.172 |
| CRP (logged) (mg/L) | -0.335 | 67.567 | 8.263 |
| Lymphocyte (%) | -17.220 | 73.250 | 8.138 |
| Mean Cell Volume | 14.773 | 54.304 | 8.221 |
| Red Cell Distribution Width | 0.869 | 55.999 | 8.194 |
| White Blood Cell Count | 2.415 | 67.914 | 8.266 |
| IL6 (logged) | 1.815 | 65.634 | 8.153 |
| TNFRI | 0.393 | 61.255 | 7.820 |
| IGF 1 | -4.704 | 73.070 | 8.088 |
| Cystatin C | 9.668 | 57.224 | 7.614 |
| NT-PROBNP (logged) | 3.373 | 52.687 | 7.242 |
| HbA1c | 0.651 | 64.245 | 8.244 |
| IL-10 | 45.991 | 66.386 | 8.233 |
| IL-1Ra | -0.039 | 68.293 | 8.265 |
| TGFB | -0.014 | 74.841 | 8.056 |
| CD4/CD8 | 0.306 | 68.061 | 8.266 |

**Supplementary Table 3. Regression Coefficients of Age and Age^2^ on Biomarkers**

|  | Age | Age^2^ |
| --- | --- | --- |
| Systolic Blood Pressure^12^ | 0.009 | -0.000 |
| Total cholesterol^2^ | -0.044*** | 0.000** |
| CMV^2^ | -0.054 | 0.001 |
| Serum Creatinine | -0.004 | 0.000 |
| Alkaline phosphatase^2^ | -0.010 | 0.000 |
| BUN^2^ | 0.000 | 0.000 |
| Peak Flow^12^ | 0.068 | -0.001** |
| Albumin | 0.020* | -0.000** |
| Fasting glucose^2^ | 0.018* | -0.000* |
| CRP (logged) | -0.022 | 0.000 |
| Lymphocyte^2^ | -0.004 | 0.000 |
| Mean Cell Volume^2^ | 0.002 | -0.000 |
| Red cell distribution width | 0.098** | -0.001* |
| White blood cell count^2^ | 0.000 | -0.000 |
| IL-6 (logged) | 0.019 | -0.000 |
| TNFRI^2^ | -0.083 | 0.002 |
| IGF 1^2^ | -0.005 | -0.000 |
| Cystatin C | -0.008 | 0.000** |
| NT-PROBNP (logged) | 0.017 | 0.000 |
| HbA1c^1^ | 0.097*** | -0.001** |
| IL-10^2^ | 0.000 | -0.000 |
| IL-1Ra^2^ | 0.186* | -0.001* |
| TGFB^2^ | -9.052* | 0.039 |
| CD4/CD8 count^2^ | 0.001 | -0.000 |
|  |  |  |
| CRP | 0.069 | -0.001 |
| IL-6 | 0.371 | -0.001 |
| NT-PROBNP | -133.605*** | 1.090*** |

***p<.001; **p<.01; *p<.05

Note: All markers are top coded at the 99%ile.

^1^2014 and 2016 combined.

^2^ Due to small but significant effect of these markers on health outcomes in regression equations, we divide these markers by 100 to visualize the effects of these markers on health outcomes in later analyses.

**Supplementary Table 4: Correlation Matrix of 24 Biomarker Variables**


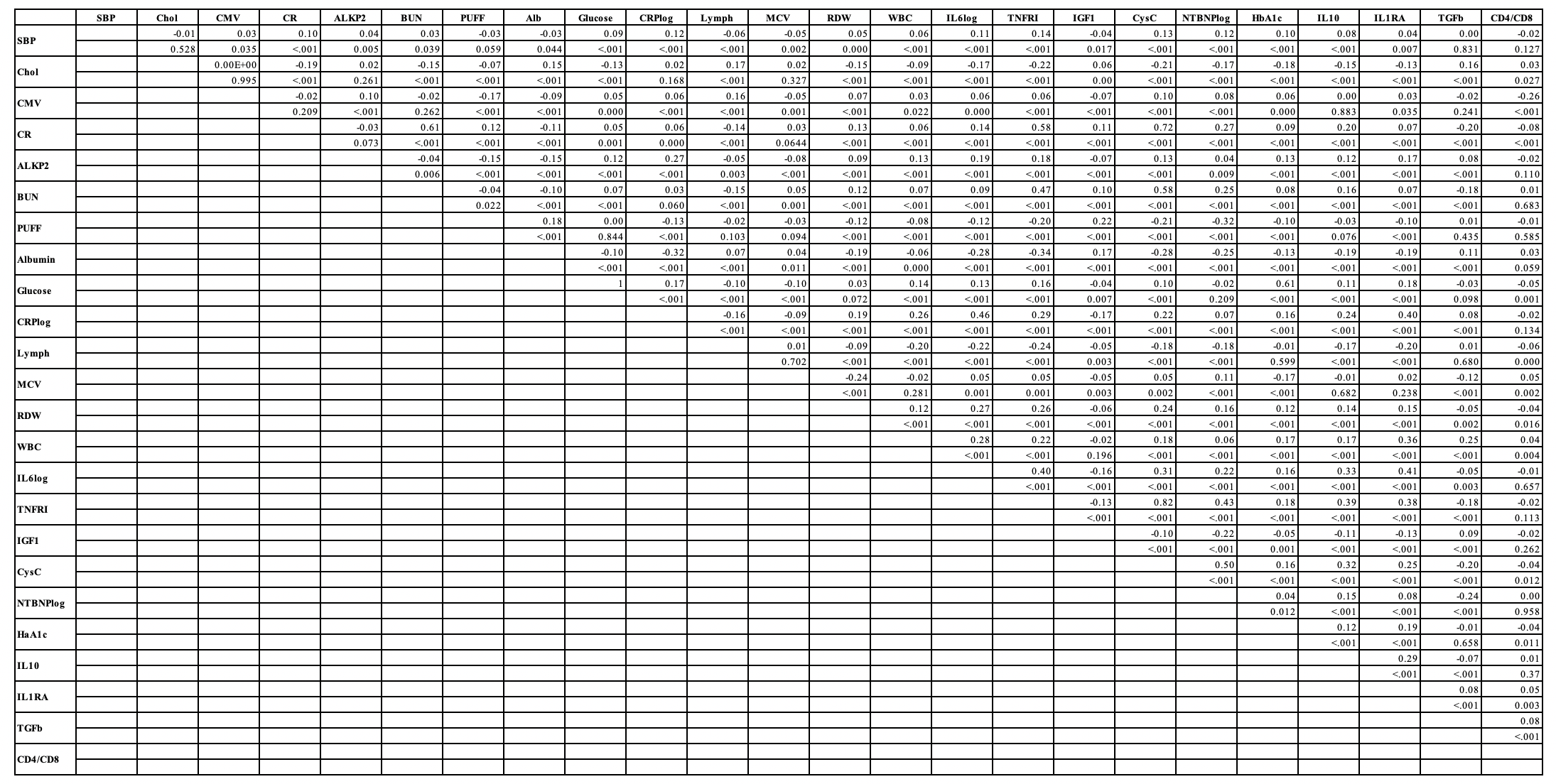


|  | Cognitive Dysfunction | Parsimonious 17  Multimorbidity | Parsimonious 10  Mortality | Parsimonious 11  ADL/IADL |
| --- | --- | --- | --- | --- |
| Systolic Blood Pressure^1^ | 0.77* | -0.11 | 2.56 |  |
| Total Cholesterol | -0.02 | -0.29*** |  | 0.12 |
| CMV | 0.04* |  |  |  |
| Alkaline Phosphatase | 0.46 |  |  | 0.33*** |
| BUN | -0.69 |  |  |  |
| Peak Flow^1^ | -1.09*** | -0.09*** | 0.57*** | -0.20*** |
| Albumin | -0.69** | 0.10 |  | -0.28*** |
| CRP (logged) | -0.06 | 0.05** |  |  |
| Lymphocyte % | 3.04*** | -0.35* | 0.08 |  |
| Mean Cell Volume | -2.30* | 0.26 | 22.77 |  |
| Red Cell Distribution Width | 0.09 | 0.02 | 1.28*** | 0.09*** |
| White blood Cell Count | 6.46 |  |  | 2.38 |
| IL6 (logged) | 0.25* | 0.01 | 1.02 | 0.05 |
| TNFRI | -0.03 |  | 1.00 |  |
| IGF 1 | -0.36* | 0.08* |  |  |
| Cystatin C | 1.09** | 0.17** | 2.08 | 0.32*** |
| NT-PROBNP (logged) | 0.09 | 0.12*** | 1.41* | 0.10*** |
| HbA1c^1^ | 0.25*** | 0.33*** |  | 0.06* |
| IL-10 | 6.93 | 0.95 | 4.69 |  |
| IL-1Ra | -0.04* | 0.02** |  |  |
| TGFB | 0.00 | -0.00 |  |  |
| CD4/CD8 | -4.56* | -1.02* |  | -0.51 |
| Age | 0.09*** | 0.01*** | 1.05*** | 0.00 |
| Female | -1.96*** | -0.19*** | 0.43*** | -0.24*** |
|  |  |  |  |  |
| Adj R^2^ | 0.2124 | 0.2712 | 0.2760 | 0.1067 |

**Supplementary Table 5: Regression Coefficients from Parsimonious Models for Health Outcomes**

^1^2014 and 2016 combined

***p<.001; **p<.01; *p<.05
